# Functioning Adrenocortical Tumors in Children-Secretory Behavior

**DOI:** 10.4274/Jcrpe.835

**Published:** 2013-03-21

**Authors:** Ali Asghar Mirsaeid Ghazi, Djafar Mofid, Mohamad-Taghi Salehian, Alireza Amirbaigloo, Khandan Zare, Bahar Jafari, Farzaneh Rahimi

**Affiliations:** 1 Research Institute for Endocrine Sciences, Shahid Beheshti University of Medical Sciences, Endocrine Research Center, Tehran, Iran; 2 Taleghani Hospital, Shahid Beheshti University of Medical Sciences, Department of General Surgery, Tehran, Iran; 3 Taleghani Hospital, Shahid Beheshti University of Medical Sciences, Department of Pathology, Tehran, Iran

**Keywords:** Adrenocortical tumors, child, virilism, feminization, Cushing’s syndrome

## Abstract

**Objective:** Adrenocortical tumors are rare childhood neoplasms. More than 95% are functional and present with virilization, Cushing’s syndrome, hypertension, or hyperestrogenism. The objective of this paper is to present the clinical, laboratory and pathological findings of this rare disease and to highlight the secretory behavior of these tumors.

**Methods:** Clinical and laboratory data of seven Iranian children and adolescents aged between 2 and 16 years with functioning adrenocortical tumors are presented. Five patients had virilization and two had Cushing’s syndrome at the time of diagnosis. In all subjects, the tumors were removed successfully by open surgery, during which a blood sample was drawn from the corresponding adrenal vein for hormonal evaluation.

**Results:** Peripheral blood evaluation revealed that in addition to the dominant hormone (testosterone in the cases presenting with virilization and cortisol in those with Cushing’s syndrome), significant amounts of other hormones were secreted from these tumors. Adrenal vein evaluation revealed that testosterone, dehydroepiandrosterone sulfate, estradiol, ?17(OH) progesterone, and cortisol were directly released from the tumor. The tumors weighed between 36-103 grams. The patients have since been followed for 5 to 20 years, and there have been no signs or symptoms of relapse in any of the patients.

**Conclusions:** The study shows that functioning adrenocortical tumors should be considered in children and adolescents presenting with hyperandrogenism, Cushing’s syndrome, or hyperestrogenism. A diagnosis of a functioning adrenocortical tumor requires surgical removal as early as possible to prevent the untoward effects of virilization or corticosteroid excess. Evaluation of adrenal vein hormones showed that the steroids are secreted directly from the tumor and peripheral conversion has little contribution to the serum levels.

**Conflict of interest:**None declared.

## INTRODUCTION

Adrenocortical tumors are rare childhood neoplasms with an annual incidence of approximately 0.3-0.5 per million children under 15 years. It is estimated that 25 new cases are seen each year in the USA. The incidence is unexpectedly high in southern Brazil, ranging from 3.4 to 4.2 per million children. It seems that high rate of mutations in the tumor suppressor gene P53 (TP53) can be a contributory factor in this unexpectedly high prevalence (1,2,3). In the majority of cases, the tumors are functional and the patients may present with virilization, Cushing’s syndrome, hypertension, hyperestrogenism or a combination of these clinical manifestations. However, they may also present with general symptoms such as abdominal pain, swelling, and discomfort. Adrenocortical tumors are usually sporadic but can sometimes be associated with tumor syndromes such as the Beckwith-Wiedemann and Li-Fraumeni syndromes (3,4,5,6,7). Low incidence, different clinical presentations, and problems in histopathologic interpretation have led to problems in precise diagnosis and treatment of the disease. The objective of this paper is to present the clinical, laboratory and pathological findings of this rare disease and to highlight the secretory behavior of these tumors by analyzing the hormonal constituents of adrenal venous effluent.

## METHODS

During the last 20 years, seven children and adolescents with functional adrenocortical tumors have been detected in the Taleghani General Hospital in Tehran, Iran. Two of the patients have been reported earlier (8,9) and an update on their follow-up is also included in this presentation. Three patients, numbers 1, 2 and 6, had been diagnosed as congenital adrenal hyperplasia (CAH) before being referred to our center and were under treatment with glucocorticoids.

The Ferriman-Gallwey score was used for scoring hirsutism and the Greulich and Pyle atlas for bone age determination (10,11). The fourth report on the diagnosis, evaluation, and treatment of high blood pressure in children and adolescents was used for defining hypertension (12). Serum testosterone, estradiol, 17(OH) progesterone, cortisol and 24-hour urine cortisol levels were measured using commercial kits. Ultrasonography and computed tomography (CT) were used for localization of the tumor. Diagnosis was based on clinical manifestations and evaluation of steroids in serum or in 24-hour urine samples.

All patients underwent open surgery. During surgery, a blood sample was collected from the effluent adrenal vein for evaluation of testosterone, estradiol, 17(OH) progesterone, and cortisol levels in all cases except one for whom it was technically impossible. In addition, a simultaneously collected peripheral vein sample was evaluated for the same hormonal profile. Histopathologic evaluation was done by 3 expert academic pathologists.

Written informed consent was obtained from the parents.

## RESULTS

**Figure 1:**
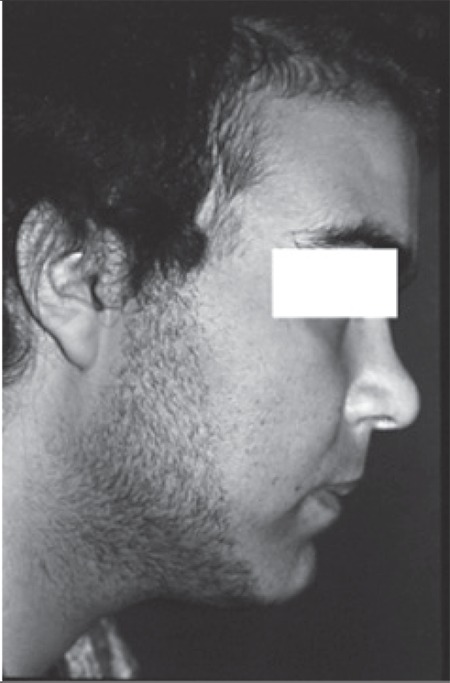
Case no 4

Clinical characteristics of the patients are shown in ?Table 1. The subjects were 5 girls and 2 boys aged 2-16 years. Duration of disease ranged from 4 months to 1 year in 6 of the patients. In case no 4, the disease was diagnosed approximately after 5 years of onset; the patient suffered from severe short stature, advanced bone age, high grade hirsutism and virilization resulting in frank emotional instability (Figure 1). Duration of patient follow-up ranged between 1 and 20 years. Signs and symptoms of virilization including temporal hair loss, hirsutism, pubic hair growth, cliteromegaly, phallic growth, voice deepening and acne were the prevailing manifestations seen in 5 of the patients. Two patients presented with Cushing’s syndrome. All patients had advanced bone age and hypertension was present in 5 of the 7 patients.

Results of laboratory evaluation of the patients at diagnosis are given in Table 2. As shown in the table, serum testosterone, dehydroepiandrosterone sulfate (DHEA-S), estradiol, 17(OH) progesterone and cortisol levels were elevated in most of the patients. Hormonal levels in adrenal vein blood samples simultaneously collected with peripheral vein samples and postoperative blood samples are shown in Table 3.

The tumors weighed 36-103 grams and were located in the left adrenal gland in all cases.

The tumors were encapsulated. A rim of compressed atrophic adrenal tissue surrounded the tumors. Microscopic evaluation, using hematoxylin-eosin staining, showed hypervascular tumors composed of lipid laden, various sized cells arranged in cords or nests. The cells had round nuclei and granular, clear or pinkish cytoplasms without mitosis. In cases 1 and 4, areas of necrosis, hemorrhage, vascular and capsular invasion were also present.

## DISCUSSION

In this paper, we attempted to portray a comprehensive clinical and laboratory picture in children and adolescent patients with functioning adrenal tumors and also to present for the first time the hormonal constituents of the effluent adrenal vein in cases with this rare disease.

Adrenocortical tumors comprise 0.3-0.5% of the neoplasms detected in patients under the age of 15 (3,4,5,6,7). Data of most patients are published as single case reports, and there are few case series addressing the problem (1,2,5,13,14). In two previous large reports on 209 and on 254 pediatric and adolescent cases, the most common presentation was virilization followed by a mixed picture of virilization and Cushing’s syndrome (15,16). The disease is more prevalent in girls, as seen in our report as well. Tumors are functionally active in 95% of cases with overproduction of androgens, corticosteroids, aldosterone and estradiol in decreasing order of frequency. Sometimes, the tumors are polyhormone secretors or their secretion pattern changes; hence, the clinical presentation may be mixed and may also change with the passage of time.

As shown in Table 2, virilization in 6 out of seven patients resulted from high serum testosterone and DHEA-S concentrations which in some cases reached levels exceeding normal values tenfold. In cases who presented with virilization, the most prominent symptoms were rapid growth, acne, deepening of voice, advancement of bone age and cliteromegaly or penile enlargement.

Cases no 1, 4 and 5 had concomitant hypersecretion of cortisol but showed no symptoms of Cushing’s syndrome. It seems that high testosterone levels obscure the usual manifestations of hypercortisolism (17,18). This finding emphasizes the importance of careful evaluation of patients for hypercortisolism in order to prevent post-surgical adrenal insufficiency which could be fatal. The high morbidity and mortality previously reported after surgical removal of these tumors might have been partly due to lack of adequate steroid supplementation during surgery (19,20).

Cases no 1, 2, and 5 were incorrectly diagnosed as CAH and were treated with corticosteroids for 2 to 9 months. This mismanagement was partly due to the higher incidence of CAH and partly because of the misconception that 17-OH progesterone is a specific marker for CAH, while the metabolite may be highly elevated in adrenocortical tumors and also in hormone-secreting testicular tumors as well (21,22). In the case reported by Hishiki et al (23), high serum 17(OH) progesterone that was used for evaluation of CAH led to diagnosis of adrenocortical tumor in a newborn. We therefore propose that adrenocortical tumors be considered in the differential diagnosis of hyperandrogenism in children. Careful consideration of testosterone, DHEA-S and androstenedione besides 17(OH) progesterone are imperative. Higher levels of testosterone and DHEA-S, lack of suppression after corticosteroid treatment, and demonstration of a lesion in adrenal gland imaging should raise the possibility of adrenocortical tumors.

Hyperestrogenism was prominent in case no 3 which had led to massive gynecomastia in the setting of Cushing’s syndrome. This patient responded well to the surgery with completion of male habitus after 1 year (Figure 2a and 2b). In addition to the case 3 who had massive gynecomastia, serum estradiol was elevated in cases 1, 3 and 4 without any symptoms and signs of feminization, a finding indicating that secretion of estradiol by adrenocortical tumors is not as unusual as is currently thought. This finding is also in accordance with the fact that functioning adrenocortical tumors have polyhormone secreting behavior, but the clinical picture is governed by the dominant hormone, namely, testosterone in virilizing syndromes and cortisol in Cushing’s syndrome. This can also be the case in patient no 6 who had Cushing’s syndrome without any virilizing symptoms despite abnormally high serum testosterone. Hence, we propose that a thorough hormonal evaluation is needed for a more accurate classification of functioning adrenocortical tumors even if there are no clinical signs or symptoms of hormone excess. As shown in Table 3, concentration of hormones in adrenal venous effluent is extremely high. As an example, the concentration of testosterone in case no 7 who was a 5.5-year-old girl was 2000 ng/dL, a figure which is nearly 2000 times in excess of the normal range.

The high ratio of the hormone levels in the adrenal vein as compared to levels in the peripheral vein points not only to the adrenal origin of the hormone (24), but also to the fact that peripheral synthesis contributed little, if any, to the hormone levels. Rapid and marked reduction of the hormones during the early postoperative week was strongly in favor of tumoral origin of the hormones and also elimination of the tumors.

In our series, all of the tumors were located in the left adrenal. It seems that adrenocortical tumors are more prevalent in the left side (13,14,25,26); although this has not always been the case (2,19,27,28). Histopathologic diagnosis was benign adrenocortical adenoma in all cases, but vascular and capsular invasion noted in cases no 1 and 4, and the weight of the tumor in case no 3 raised a possibility of malignant nature of these tumors; however, the long-term post-surgical follow-up of 2 to 12 years showed the benign nature of these neoplasms.

Our findings are in accordance with data reported by other investigators who showed that in childhood functioning adrenocortical tumors, the size and weight of the tumors are more important factors in determining the benign or malignant nature of the tumor. Indeed, tumors weighing less than 100 grams have a benign nature and a favorable prognosis (29,30,31,32,33).

In some studies, functioning adrenocortical tumors are reported to have a good prognosis (13). This finding may be just due to earlier diagnosis because of earlier clinical manifestations secondary to hormone over secretion (19). There has been no significant difference in prognosis between functional and nonfunctional tumors in other series (19,25,34). Some but not all studies have shown that prognosis is better in children in comparison to adults (2,13,16,19,28,30,34). Age may not be a major prognostic determinant when other more important factors such as size and weight of the tumor are taken into account.

In conclusion, this study supports the notion that childhood and adolescence adrenocortical tumors usually present with clinical manifestations that should be immediately diagnosed and surgically treated to avoid untoward complications. Thorough evaluation of hormonal profile, irrespective of hormone-related clinical manifestations, will reveal the usually multi-hormonal secreting nature of the tumors. Assessment of adrenal venous effluent hormones has shown that hormones are directly secreted from the tumor and peripheral conversion has little contribution to peripheral hormone levels.

**Acknowledgement**

The authors wish to thank the patients and their parents for making the study possible.

## Figures and Tables

**Table 1:**
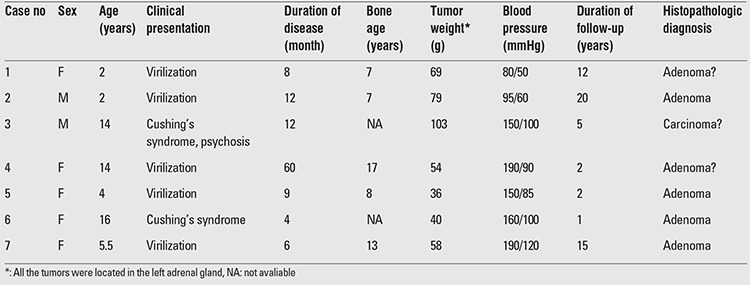
Clinical characteristics of cases with adrenocortical tumor

**Table 2:**
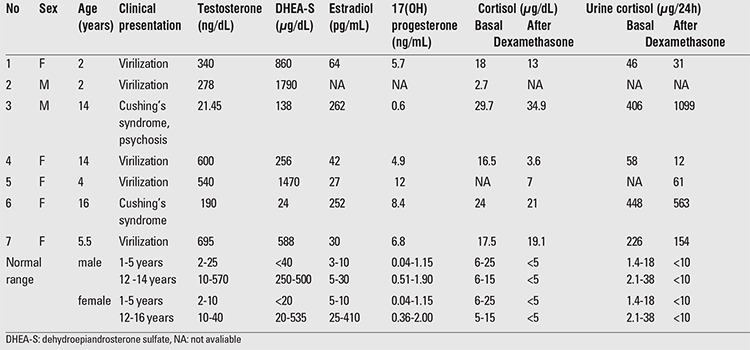
Clinical characteristics, serum and urine hormonal levels of the cases with adrenocortical tumor

**Table 3:**
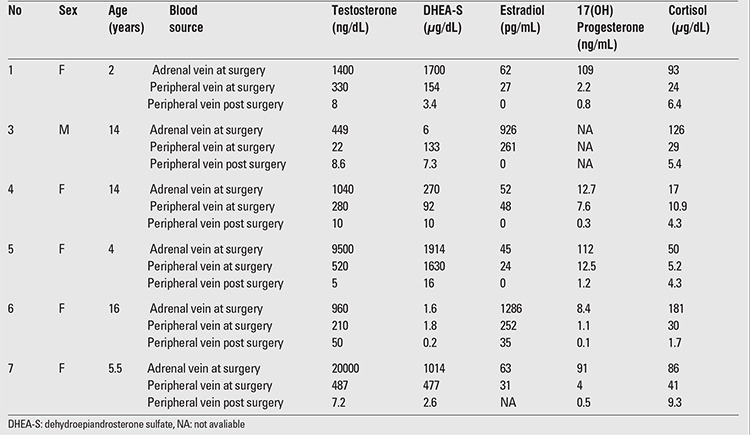
Hormone levels in adrenal and peripheral vein samples collected simultaneously at surgery and peripheral vein samples obtained one week after surgery

**Figure 2a:**
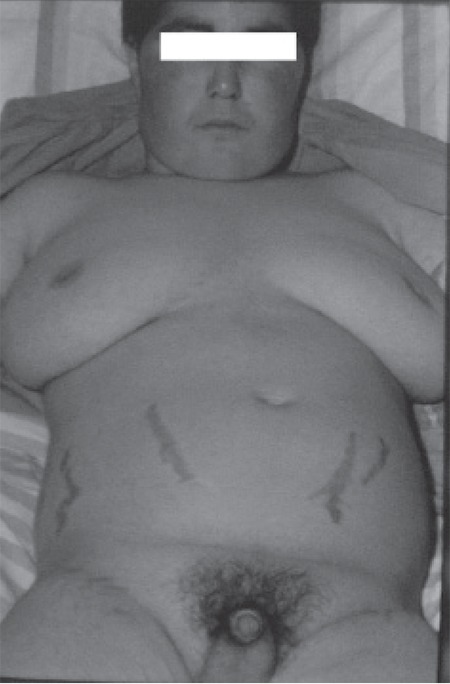
Case no 3 before surgery

**Figure 2b:**
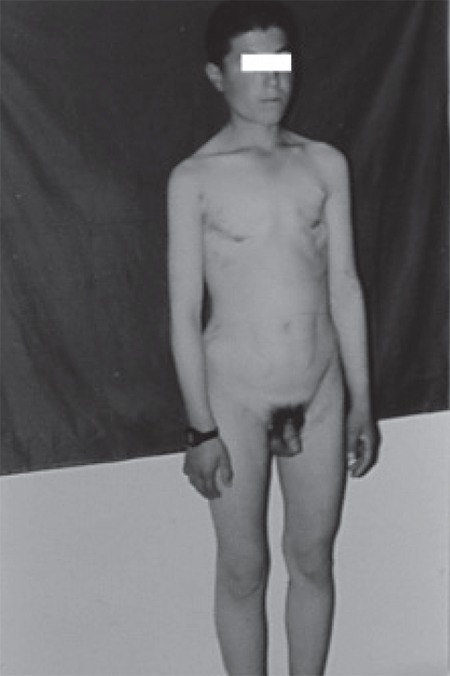
Case no 3 after surgery
